# Extended‐spectrum β‐lactamase‐producing Enterobacteriaceae from ready‐to‐eat foods: Genetic diversity and antibiotic susceptibility

**DOI:** 10.1002/fsn3.3513

**Published:** 2023-06-27

**Authors:** Yao Liu, Xinghua Chen, Junwei Luifu, Jing Zhao, Xujun He, Tengfei Xie

**Affiliations:** ^1^ Guangdong Eco‐engineering Polytechnic Guangzhou China

**Keywords:** antibiotic resistance, ERIC‐PCR, ESBL‐producing Enterobacteriaceae, MLST

## Abstract

Ready‐to‐eat (RTE) foods are widely marketed in China and are important components of everyday diet. In this study, a total of 2000 RTE food samples were analyzed, 252 (12.60%) of which were positive for Enterobacteriaceae, and 48 were identified as containing extended‐spectrum β‐lactamase (ESBL)‐producing *Escherichia coli* isolates. Furthermore, the antimicrobial resistance patterns of these isolates to 14 antimicrobial agents revealed that most isolates were resistant to aminoglycosides and β‐lactam antibiotics. The TEM‐type gene was prevalent in our isolates (79.17%). The isolates (*n* = 48) were classified into three clusters based on the ERIC‐PCR results. Forty‐eight sequence types were found without duplicates, revealing genetic variation and relatedness among isolates. Thus, the results demonstrated the presence of ESBL‐producing *Enterobacteriaceae* in Chinese RTE foods. The results of this study provide insights into the spread of antibiotic‐resistant strains and improve understanding of microbial risks.

## Graphical abstract

As RTE food is a common and popular food choice in China, these findings also highlight the importance of establishing an accurate monitoring plan to track and control the further spread of ESBL clones.

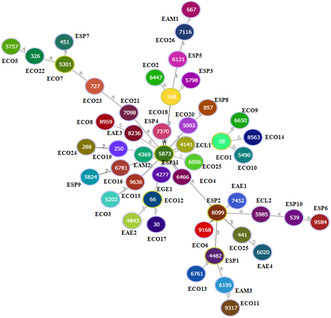

## 1 INTRODUCTION

The rapid emergence and spread of antibiotic‐resistant bacteria—in particular, extended‐spectrum β‐lactamase (ESBL)‐producing Enterobacteriaceae—is a serious threat to public health and needs to be controlled effectively (Kebede et al., 2022; Xu et al., 2018). ESBL enzymes can hydrolyze β‐lactam antibiotics, including third‐generation cephalosporins, such as ceftazidime (CAZ), ceftriaxone, and cefotetan, which makes these enzymes important tools for drug resistance mechanisms (Brolund et al., 2019; Gupta et al., 2022). Although ESBL‐producing Enterobacteriaceae are known to occur in many communities of food‐producing animals, such as chickens (Randall et al., 2020) and cattle (Yang et al., 2018), studies on the presence of ESBL‐producing bacteria in ready‐to‐eat (RTE) foods have rarely been conducted in China.

RTE foods are products that are processed after the raw materials have been marinated, smoked, baked, or fried. Generally, these foods can be divided into two categories: special salads and fried foods. RTE foods are easy to prepare and taste delicious, according to consumers across the country. Every day, the majority of China's markets, restaurants, and hotels are supplied with rich RTE foods. As these are consumed without further processing, maintaining hygiene during all food‐processing operations is essential to ensure public health. Nevertheless, microbiological hazards pose challenges to the RTE food industry (Cole & Singh, 2018; Iseppi et al., 2018; Thirupathi & Krishnan, 2020), and hence, it is necessary to study antibiotic‐resistant bacterial strains present in RTE foods.

Excessive dependence on antimicrobial agents is inevitable, owing to their unrestricted access and negligent supervision. Compared with other classes of drugs, β‐lactam antibiotics are relatively inexpensive, safe, and have fewer side effects, making them the most used antibiotics in human medicine. ESBL‐producing bacteria are resistant to penicillin and new‐generation cephalosporins (Dikoumba et al., 2021). Therefore, it is necessary to monitor antibiotic‐resistant phenotypes. The spectrum of prevalent plasmid‐mediated enzymes responsible for this resistance has changed from predominantly *TEM*‐ and *SHV*‐type to *CTX*‐*M*‐ and *OXA*‐type beta‐lactamases (Livermore et al., 2006). These genes are encoded by plasmids and usually express broad‐spectrum β‐lactamases that impart resistance to aminopenicillins and cephalosporins. Therefore, checking for the presence of these four genes is an effective method to determine whether a bacterial strain is resistant.

Molecular typing methods, such as pulsed‐field gel electrophoresis (PFGE) (Kao et al., 2016) and repetitive extragenic palindromic sequence‐based polymerase chain reaction (PCR) (Valenza et al., 2017), have proven to be effective tools for providing genetic information and detecting contamination in epidemiological studies. Many pathogens can be subtyped using enterobacterial repetitive intergenic consensus (ERIC)‐PCR, which makes use of highly conserved consensus sequences between repetitive intergenic sequences (Gonçalves et al., 2016). Additionally, multilocus sequence typing (MLST) based on sequence analysis of selected housekeeping genes is becoming an important method for studying strain evolution and epidemiology, owing to its high repeatability (Hu et al., 2022), and is a preferred method in global epidemiological research.

ESBL‐producing Enterobacteriaceae, harmful to human health, can be found in RTE foods. In this study, phenotyping and genotyping methods were used to determine the correlations between these isolates. Diverse strains were present in RTE foods, and the findings of this study can assist in evaluating the microbial profiles in RTE foods and ensuring food safety.

## 2 MATERIALS AND METHODS

### 2.1 Sample collection and pathogen detection

A total of 252 strains of Enterobacteriaceae were isolated from 2000 RTE food samples in Guangdong, China, in 2021. To isolate *Escherichia coli*, 25 g of foodstuff was transferred to an aseptic bag and mixed with 225 mL of Butterfield's phosphate‐buffered solution. The sample was homogenized for 2 min at 230 rpm using a stomach machine and diluted 10‐fold. The diluent was inoculated into fermentation tubes containing lactose broth and incubated at 37°C for 24–48 h. A loopful of the suspension extracted from positive cultures (those showing lactose fermentation and gas production) was smeared onto CHROMagar *E. coli* agar plates and cultured at 37°C for 18–24 h. Finally, one colony from each plate was selected, analyzed, and identified using API 20E (bioMérieux) (Qinghua et al., 2018).

### 2.2 Confirmatory tests for ESBL‐producing bacteria

Disc diffusion tests were used to screen ESBL‐producing bacterial strains. Mueller–Hinton agar was swabbed with a suspension of pure culture (0.5 McF) and loaded with antibiotic discs.

Previous studies have shown that CTX (30 μg) and CAZ (30 μg) can be used to screen for ESBL‐producing Enterobacteriaceae strains (Maravic et al., 2015). A double‐disc synergy test (DDST), according to the Clinical and Laboratory Standards Institute (CLSI) guidelines (2019), using CTX and CAZ, with and without clavulanic acid, was used to assess the presence of ESBLs (Mast Diagnostics). Compared to the area diameter of the antimicrobial agent tested alone, the area diameter of antimicrobial agents tested in combination with clavulanic acid increased by over 5 mm. These results confirmed the presence of ESBL‐producing organisms.

### 2.3 Antimicrobial susceptibility testing

In accordance with the 2019 CLSI guidelines, the disc diffusion method was used to evaluate the sensitivity of the following antibiotics: aminoglycosides (gentamicin, GEN, 120 μg; kanamycin, KAN, 30 μg; and streptomycin, SM, 300 μg), β‐lactams (ampicillin, AMP, 10 μg; amoxicillin, AMC, 20 μg; cephazolin, 30 μg; imipenem, IPM, 10 μg; meropenem, MEM, 10 μg; and piperacillin, PIP, 100 μg), macrolides (azithromycin, AZM, 15 μg and erythromycin, ERY, 15 μg), quinolones (ciprofloxacin, CIP, 5 μg and nalidixic acid, NA, 30 μg), and tetracyclines (minocycline, MIN, 30 μg and tetracycline, TET, 30 μg). All samples were obtained from Oxoid Ltd. *Staphylococcus aureus* ATCC 25923 and *E. coli* ATCC 25922 were used as control strains. Precision calipers were used to measure the zones of inhibition with an accuracy of 0.01 mm. Microbial strains that showed resistance to at least three antimicrobial agents were considered multidrug resistant (Qinghua et al., 2018).

### 2.4 Detection of ESBL genes

All strains showing positive phenotypes in the ESBL screening test by DDST were evaluated by PCR to check for the presence of β‐lactamase‐encoding genes *CTX‐M*, *OXA*, *SHV*, and *TEM* (Dierikx et al., 2012; Kebede et al., 2022). Genomic DNA was extracted from the positive strains using Bacterial Genomic DNA Purification Kits (Dongsheng Biotech), according to the manufacturer's instructions. The concentration of genomic DNA was measured based on the absorbance at 260 nm using a NanoDrop® ND‐1000 Ultraviolet–visible Spectrophotometer (Thermo Fisher Scientific).

### 2.5 Genetic relatedness of collected isolates

ERIC‐PCR analysis was used to investigate genetic relatedness among *E. coli* isolates. The reaction system used was as follows: the PCR mixture (total 25 μL) was composed of 2× Long Taq Mix (Dongsheng Biotech), 12.5 μL; primers (5'‐ATGTAAGCTCCTGGGGATTCAC‐3' and 5'‐AAGTAAGTGACTGGGGTGAGCG‐3'), 0.5 μM; and template DNA, 50 ng. The amplification was performed under the following temperature curve: initial denaturation at 95°C for 5 min; 36 cycles including those at 95°C for 1 min, 46°C for 1 min, and 72°C for 2 min; and, finally, 72°C for 8 min. The ERIC‐PCR products were separated and detected using 2.0% agarose gel electrophoresis with Gold View (0.005% v/v). The resulting images were saved in TIFF format and used for further analyses.

The bands on the ERIC‐PCR fingerprint patterns were analyzed using BioNumerics 7.6 MLST analysis performed on the MLST website and database (https://pubmlst.org/escherichia/). Seven housekeeping genes (*adk*, *fumC*, *gyrB*, *icd*, *mdh*, *purA*, and *recA*) were amplified and sequenced using PCR. The PCR conditions were as follows: denaturation at 95°C for 5 min; 30 cycles including 95°C for 1 min, 60°C for 1 min, and 72°C for 1 min; and, finally, 72°C for 10 min. All primers were obtained from the website and were synthesized using a BGI instrument. PCR products were sequenced using a BGI instrument. All sequences were analyzed online to determine the number of alleles and confirm the sequence types (STs). The evolutionary tree was constructed based on seven loci sequences and was analyzed using BioNumerics 7.6 (Applied Maths).

## 3 RESULTS

### 3.1 Identification of ESBL‐producing *Enterobacteriaceae*

An overview of the 2000 RTE samples showed that 252 (12.60%) among them were positive for Enterobacteriaceae. According to the results of the disc diffusion method, of these 252 samples, 78 (30.95%) showed resistance or moderate resistance to CTX and/or CAZ. The selected resistant samples were subjected to preliminary screening for ESBL‐producing strains. Using DDST, 48 (42.11%) samples were found to contain ESBL‐producing bacterial strains. The results showed that the prevalence of *E. coli* was the highest, followed by that of the *Enterobacter* species *E. aerogenes*, *E. cloacae*, *E. gergoviae*, and *E. amnigenus*, which were identified in relatively fewer numbers (Table 1).

**TABLE 1:** Prevalence and levels of ESBL‐producing Enterobacteriaceae strains in samples.

Species	No.	Number of ESBL‐producing strains (%)	
		CTX and CAZ resistant	Confirmed by DDST
*E. coli*	114	56 (49.12)	48 (42.11)
*Enterobacter spp*	45	6 (13.33)	/
*E. aerogenes*	33	5 (15.15)	/
*E. cloacae*	27	4 (14.81)	/
*E. gergoviae*	17	2 (11.76)	/
*E. amnigenus*	16	5 (31.25)	/
Total	252	78 (30.95)	48 (19.05)

### 3.2 Sensitivity analysis of ESBL‐producing strains

Antimicrobial susceptibility tests for the 48 ESBL‐producing isolates were performed using the standard disc diffusion method. Analysis of β‐lactam sensitivity patterns revealed that the 48 ESBL‐producing strains showed high resistance to AMP (91.67%) and low resistance to carbapenems (Table 2). The results also showed that the strains expressed low‐to‐moderate resistance to IPM (4.17%) and MEM (6.25%). However, more than half of the strains were resistant to other antibiotics, including aminoglycosides, macrolides, and quinolones, such as GEN (39.58%), KAN (43.75%), SM (50.00%), AZM (56.25%), and CIP (52.08%). Fewer strains were resistant to tetracycline antibiotics, such as MIN (18.75%) and TET (22.92%). Additionally, except for one strain that was resistant to only one antibiotic and one strain that showed resistance to two antibiotics, all other strains showed a multidrug resistance phenotype (12 isolates). The multidrug resistance phenotype was resistant to at least three types of antibiotics.

**TABLE 2:** Number of antibiotic‐resistant ESBL‐producing Enterobacteriaceae isolates.

Antimicrobial agents	No. (%) of R	No. (%) of I	No. (%) of S
Aminoglycosides			
Gentamicin (GEN)	19 (39.58)	6 (12.50)	23 (47.92)
Kanamycin (KAN)	21 (43.75)	7 (14.58)	20 (41.67)
Streptomycin (SM)	24 (50.00)	5 (10.42)	19 (39.58)
β‐lactams			
Ampicillin (AMP)	44 (91.67)	1 (2.08)	3 (6.25)
Amoxicillin (AMC)	20 (41.67)	4 (8.33)	24 (50.00)
Imipenem (IPM)	2 (4.17)	3 (6.25)	43 (89.58)
Meropenem (MEM)	3 (6.25)	3 (6.25)	42 (87.50)
Piperacillin (PIP)	5 (10.42)	1 (2.08)	42 (87.50)
Macrolides			
Azithromycin (AZM)	27 (56.25)	9 (18.75)	12 (25.00)
Erythromycin (ERY)	19 (39.58)	7 (14.58)	22 (45.83)
Quinolones			
Ciprofloxacin (CIP)	25 (52.08)	6 (12.50)	17 (35.42)
Nalidixic acid (NA)	23 (47.92)	5 (10.42)	20 (41.67)
Tetracyclines			
Minocycline (MIN)	9 (18.75)	4 (8.33)	35 (72.91)
Tetracycline (TET)	11 (22.92)	7 (14.58)	30 (62.50)

Abbreviations: I, intermediate resistance; R, resistant; S, susceptibility.

### 3.3 Characterization of β‐lactamase genes

One or more β‐lactamase‐encoding genes were detected in the 48 isolated strains. TEM‐type β‐lactamase was prevalent in the strains (79.17%), followed by SHV (68.75%), CTX‐M (35.42%), and OXA (20.83%). SHV combined with TEM‐β‐lactamase was found to be the most common type of ESBL. SHV and TEM were detected in 10.42% and 8.33% of the samples, respectively. Two isolates tested positive for all four β‐lactamase genes–strains ECO2 and ESP6 (Table S1).

### 3.4 ERIC‐PCR analysis

**FIGURE 1:**
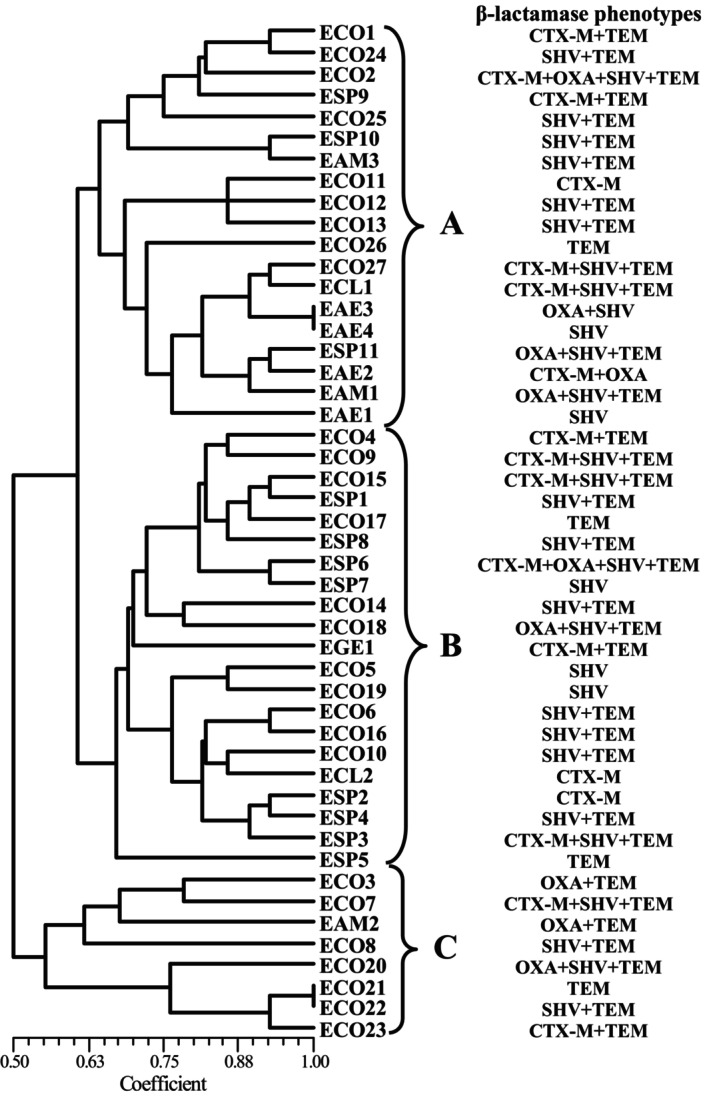
ERIC‐PCR DNA fingerprint analysis of ESBL‐producing Enterobacteriaceae isolates.

The results of ERIC‐PCR analysis of the 48 isolates are shown in Figure 1. Approximately 4–10 amplification bands in the ERIC‐PCR results were found, ranging from 130 to 5000 bp. Based on the relative similarity coefficient, the strains were classified into three clusters, designated A, B, and C. It was found that the same strains clustered closely.

### 3.5 MLST analysis

**FIGURE 2:**
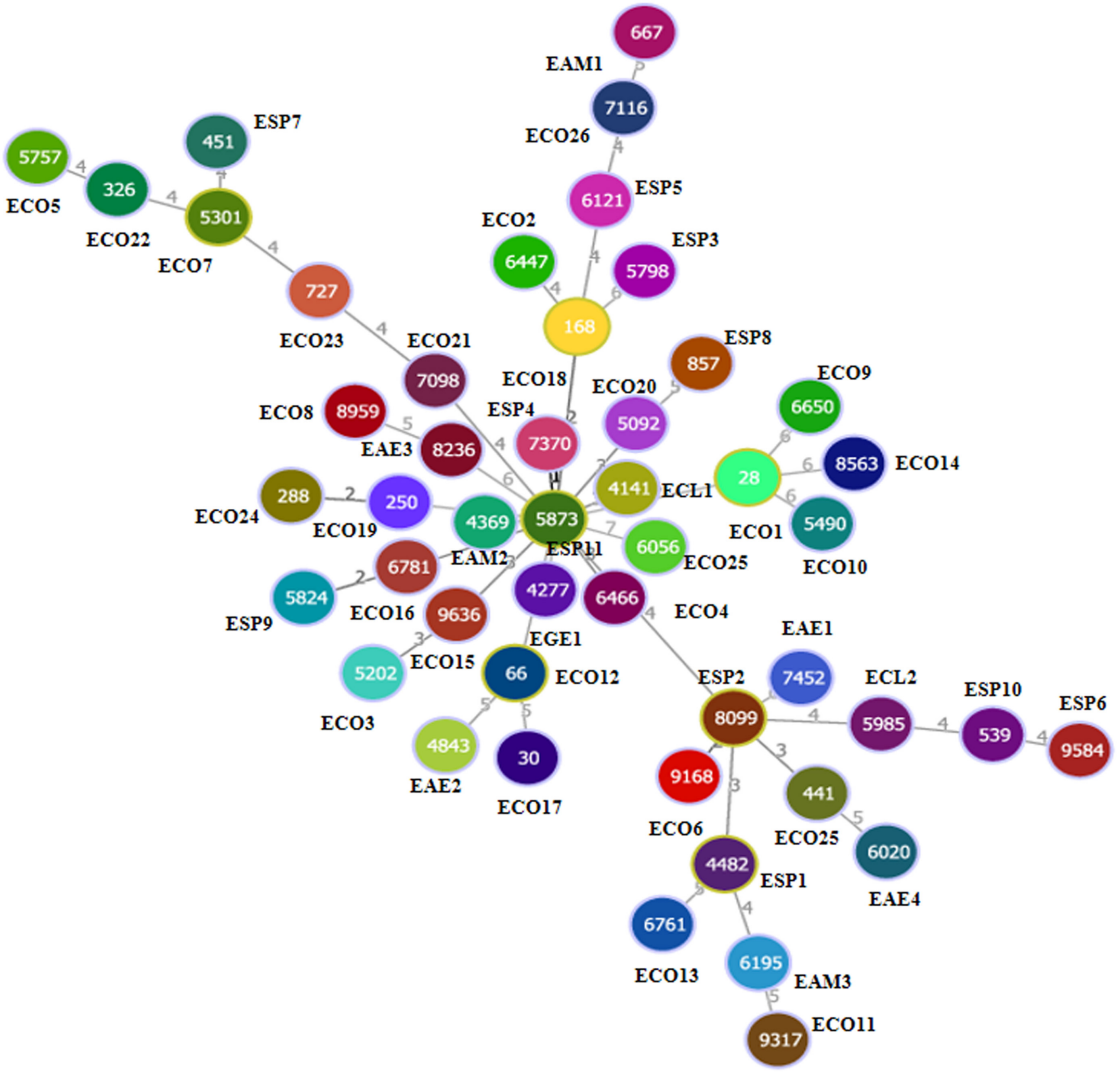
MLST minimum evolution tree of ESBL‐producing Enterobacteriaceae isolates.

Seven housekeeping gene sequences were used for MLST analysis. The sequences of *Escherichia* isolates were uploaded to the MLST database and assigned numbers for alleles and STs. Overall, 48 STs were obtained without any new locus. According to the results, the number of each MLST locus was as follows: *adk*: 20, *fumC*: 27, *gyrB*: 26, *icd*: 25, *mdh*: 25, *purA*: 24, *recA*: 20. The minimum evolutionary tree composed of the tandem sequences of each allele is shown in Figure 2. The sequence ST5873 (ESP11) was in the middle and was related to most strains.

## 4 DISCUSSION

The high prevalence of ESBL‐producing Enterobacteriaceae in developing countries is a serious concern and is attributed to the widespread practice of self‐treatment, hospital crowding, the absence of antibiotic prescription guidelines, and poor sanitation (Hoda et al., 2022). RTE foods, such as roasted poultry, pot‐stewed meat, and vegetable salad, are popular elements of Chinese dinners. Various food‐borne pathogens can be detected in these RTE foods, which do not need any further treatment before eating; therefore, the risk of disease among consumers is high (Shala et al., 2018). Most RTE products contain opportunistic bacteria that are not directly associated with human diseases; however, ESBL‐producing Enterobacteriaceae pose a great risk to humans (Peirano & Pitout, 2019) as Enterobacteriaceae are widespread and ESBL‐producing Enterobacteriaceae are often not detected. We analyzed 252 strains of bacteria from Chinese RTE foods and isolated 78 (30.95%) CTX‐ and/or CAZ‐resistant Enterobacteriaceae. The results show that 48 out of 238 RTE products yielded ESBL‐producing Enterobacteriaceae. This incidence rate is slightly higher than that reported in a previous study (Nueesch‐Inderbinen et al., 2018). In Italy, resistance to β‐lactam antibiotics was observed in 44 out of 312 Gram‐negative strains (14.1%) (Iseppi et al., 2018). However, only a few studies on ESBL‐producing Enterobacteriaceae have been conducted in China. Qinghua et al. (2018) investigated the presence of ESBL‐producing Enterobacteriaceae in retail foods, including 72 RTE food samples, and reported an incidence rate of 11.1%. The higher value obtained in the present study may be attributed to the increased antibiotic use in recent years. RTE foods, which are convenient and healthy, meet consumer needs. However, few people opt to process them before eating; therefore, the identification of ESBL‐producing Enterobacteriaceae in RTE foods is essential to ensure food safety.

To increase aquaculture production, farmers use various antibiotics to prevent and treat pathogenic bacterial infections in aquatic products (Saidani et al., 2018). This adds to the increasing antibiotic resistance of Enterobacteriaceae and has become a recent research focus. Antimicrobial‐ and multidrug‐resistant Enterobacteriaceae are frequently studied (Kebede et al., 2022), and multiple β‐lactamases, such as ESBLs, cephalosporinases, penicillinases, and metallo‐β‐lactamases, that contribute to resistance have been identified (Pitout, 2012). Among these, ESBL‐producing bacteria are particularly important as their resistance stems from enzymes involved in ESBL production. These enzymes have strong antimicrobial activity and can hydrolyze even third‐generation cephalosporins and penicillin (Dikoumba et al., 2021). In this study, approximately 50% of the ESBL‐positive strains were found to be resistant to aminoglycosides (GEN, KAN, and SM), β‐lactams (AMP and AMC), macrolides (AZM and ERY), and quinolones (CIP and NA). This phenomenon is consistent with that reported in a Japanese study (Yokoyama et al., 2018). In Tunisia, resistance was observed principally against TET, NA, GEN, and sulphonamides such as trimethoprim. Antimicrobial susceptibility in Antananarivo in Madagascar was more serious, particularly toward GEN (87.7%), tobramycin (93.8%), CIP (69.3%), and trimethoprim–sulfamethoxazole (100%) (Rakotonirina et al., 2013). Currently, carbapenems are considered the most reliable antibiotics for treating ESBL‐producing Enterobacteriaceae. Previous studies have reported that a few ESBL‐positive strains are resistant to carbapenems, even in clinical settings (Yang et al., 2018). In contrast, only 4.17% and 6.25% of strains showed resistance to IPM and MEM, respectively, in the present study. It is important to assess the changes in the antimicrobial susceptibility profiles of these strains because RTE foods pose health risks when consumed without cooking.

ESBLs include TEM‐, SHV‐, OXA‐, and CTX‐M‐type enzymes. SHV‐ and TEM‐type ESBLs may contribute to third‐generation cephalosporin resistance. With the increasing use of different antibiotics, the prevalence of CTX‐M‐type ESBLs has exceeded that of TEM‐ or SHV‐type enzymes, and these have become the most predominant type since the year 2000 (Yohei et al., 2012). As the rate of ESBL production varies greatly among bacterial species worldwide, trends in ESBL gene types will also change over time. For example, production of TEM‐type ESBL in *E. coli* was widespread in China, followed by that of SHV and CTX‐M. However, in Canada, SHV‐type is widespread, followed by those of TEM and CTX‐M. To date, CTX‐M has been reported to be the most widely distributed ESBL‐producing gene in India (Dikoumba et al., 2021). The results of the present study revealed that TEM (79.17%) is the most predominant ESBL gene in Chinese retail food samples, which is consistent with the results of another study (Yang et al., 2018). The coexistence of these four genes was observed in two isolates in the present study. The content of these three harboring genes was higher than that of these genes in North India, where only 6.45% of the isolates were observed to possess these genes. According to another study from Brazil on ESBL‐producing *Klebsiella* and *Enterobacter* spp., 52.9% of *K. pneumonia* and 10.3% of *E. cloacae* carried the *TEM* gene. These values are also lower than those of the present study. The present study also showed a higher prevalence of the CTX‐M gene (35.42%), albeit lower than that reported in Southern Brazil (90.32%) (Jena et al., 2017). The results showed that plasmids can carry ESBL β‐lactamase genes and can also spread horizontally in Enterobacteriaceae, which is the cause of the high incidence of multidrug resistance.

Molecular subtyping is widely used in the epidemiological analysis of pathogens. It also identifies ESBL‐producing Enterobacteriaceae by PFGE and reveals genetic diversity (Philippe et al., 2022). Compared to PFGE, ERIC‐PCR can provide discriminatory values and is easy to perform. Based on the ERIC‐PCR results, the strains were divided into three clusters with 0.63 similarity. Clustering based on the ERIC‐PCR results was inconsistent with the source or pattern of antibiotic resistance. However, it was found that strains of the same species were close to each other (Cluster C). MLST is a good typing method owing to its high reproducibility, as shown by the sequencing of seven housekeeping genes. This method has also been widely used for the sequence analysis of Enterobacteriaceae. Some reports have shown that highly uncommon sequence types can occur in the environment, animals, or humans too (Saidani et al., 2018). In this study, the strains were divided into three clusters. Up to 48 STs were identified in 48 isolates; a high proportion of STs was found, indicating a high degree of diversity among ESBL‐producing Enterobacteriaceae isolates. These results suggest that the Enterobacteriaceae strains found in RTE foods differ from other strains. ERIC‐PCR and MLST have simultaneously confirmed the genetic diversity of these strains (Martischang et al., 2020).

This study comprehensively identified the antibiotic resistance phenotype and molecular subtype of ESBL‐producing bacteria in Chinese RTE foods. The pattern of antibiotic resistance showed that drug resistance is extensive, and the bacterial strains are resistant to some clinical antibiotics too, including MEM and PIP. ERIC‐PCR and MLST studies revealed genetic diversity. As RTE foods are becoming a common food choice in China, these results highlight the importance of establishing precise methodologies for tracking and controlling further spread of ESBL clones.

## AUTHOR CONTRIBUTIONS

**Yao Liu:** Writing – original draft (equal). **Xinghua Chen:** Writing – original draft (equal). **Junwei Luifu:** Writing – review and editing (equal). **Jing Zhao:** Writing – review and editing (equal). **Xujun He:** Writing – review and editing (equal). **Tengfei Xie:** Methodology (equal); writing – original draft (equal).

## CONFLICT OF INTEREST STATEMENT

There is no conflict of interest to declare.

## Supporting information


Table S1.

## Data Availability

The data that support the findings of this study are openly available in figshare at https://doi.org/10.1002/fsn3.3513, reference number FSN3‐2022‐11‐1578.
